# Spontaneous Intracranial Hypotension Treated with a Targeted CT-Guided Epidural Blood Patch

**DOI:** 10.1155/2016/9809017

**Published:** 2016-02-11

**Authors:** Inês Correia, Inês Brás Marques, Rogério Ferreira, Miguel Cordeiro, Lívia Sousa

**Affiliations:** ^1^Department of Neurology, Centro Hospitalar e Universitário de Coimbra, Coimbra, Portugal; ^2^Department of Internal Medicine, Centro Hospitalar e Universitário de Coimbra, Coimbra, Portugal; ^3^Department of Neuroradiology, Centro Hospitalar e Universitário de Coimbra, Coimbra, Portugal

## Abstract

Spontaneous intracranial hypotension (SIH) is an important cause of new daily persistent headache. It is thought to be due to spontaneous spinal cerebrospinal fluid (CSF) leaks, which probably have a multifactorial etiology. The classic manifestation of SIH is an orthostatic headache, but other neurological symptoms may be present. An epidural blood patch is thought to be the most effective treatment, but a blind infusion may be ineffective. We describe the case of a young man who developed an acute severe headache, with pain worsening when assuming an upright posture and relief gained with recumbency. No history of previous headache, recent cranial or cervical trauma, or invasive procedures was reported. Magnetic resonance imaging showed pachymeningeal enhancement and other features consistent with SIH and pointed towards a cervical CSF leak site. After failure of conservative treatment, a targeted computer tomography-guided EBP was performed, with complete recovery.

## 1. Introduction

Spontaneous intracranial hypotension (SIH) is a known cause of new daily persistent headache. Although the classic feature is an orthostatic headache, other symptoms related to the pathophysiology of SIH, such as neck pain or stiffness, nausea, vomiting, imbalance, or visual complaints, may also be present with variable clinical severity. Conservative treatment is usually attempted first; however, this approach is unsuccessful in many patients. In these cases, an epidural blood patch (EBP) is usually considered. It is common to perform a blind EBP, but it can be ineffective and several trials may be necessary to achieve success, with some patients being refractory to this approach. A targeted CT-guided EBP is a more recent treatment option, with a higher success rate reported, but technical expertise is needed.

## 2. Case Report

We report the case of a 34-year-old man who presented to our emergency department complaining of a severe, diffuse, and throbbing headache. The headache had started two weeks before and shown a progressive worsening with a gradual increase in intensity during this period. The pain was immediately exacerbated when the patient assumed an upright position but was partially relieved after approximately five minutes of recumbency. Common analgesics brought no relief. The patient also complained of nausea, vomiting, photophobia, the sensation of neck stiffness from headache onset, and episodic double vision during the previous two days. He denied having a history of previous headache, recent head or neck trauma, or invasive procedures. There was a personal medical history of psoriasis and asthma, but he was asymptomatic at the time. He attended the gym on a daily basis and he had played tennis on the day before the headache onset. On the fourth day of symptoms, the patient resorted to going to the emergency department of a local hospital, where a brain computerized tomography (CT) was requested. The exam did not show any abnormal findings, and the patient was discharged with symptomatic analgesics.

The physical and neurological examination on admission to our emergency department were remarkable only for a left sixth nerve paresis and severe photophobia.

**Figure 1:**
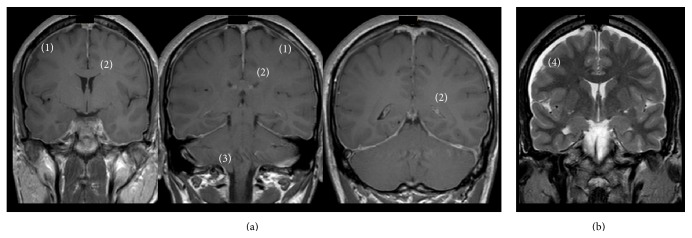
(a) T1-weighted brain MRI scan with gadolinium showing diffuse pachymeningeal enhancement (1), decrease in ventricle size (2), and cerebellar tonsil descent (3). (b) T2-weighted brain MRI scan showing subdural fluid collections (4).

A brain magnetic resonance imaging (MRI) scan was performed and revealed diffuse pachymeningeal enhancement, a decrease in ventricle size, cerebellar tonsil descent, and subdural fluid collections, findings which were compatible with SIH (Figure 1).

**Figure 2:**
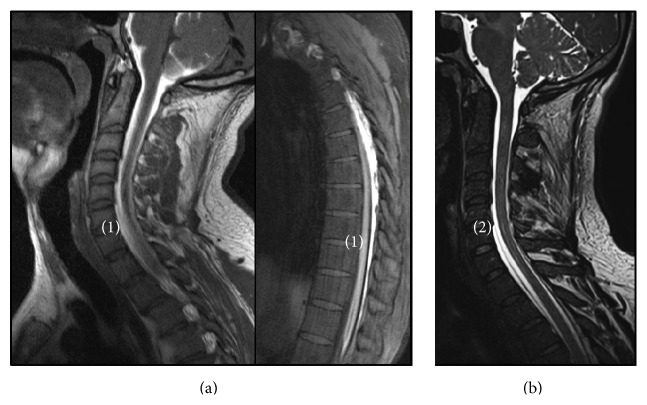
(a) T1-weighted spine MRI scan after intrathecal administration of gadolinium: sagittal views showing extra-arachnoid cerebrospinal fluid collections enhanced with gadolinium (1). (b) T2-weighted spine MRI scan showing subdural fluid collections and site of leak (2).

MRI myelography was then requested. A low cerebrospinal fluid (CSF) opening pressure (5 cm H_2_O) was confirmed during the procedure and a spread of CSF in the epidural space was observed, extending from L3 to the cervical region, entering the intrathecal space at C7, suggesting a CSF leak located at this level (Figure 2).

**Figure 3:**
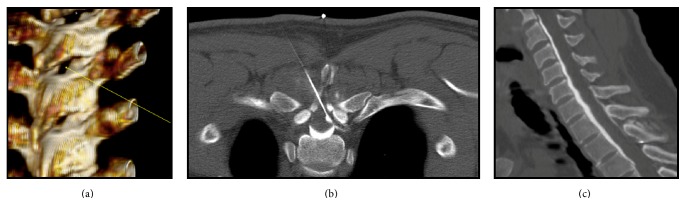
(a) Spine CT-scan three-dimensional simulation site for targeted EPB. (b) Targeted CT-guided EBP. (c) Spine CT-scan showing contrasted blood injected in EBP.

A conservative approach was initially tried, consisting of bed rest, hydration, analgesia, and caffeine intake. After two weeks without clinical improvement, it was decided to proceed with an EBP. Since the CSF leak was located at a cervical level and the literature suggests that these cases are less likely to resolve with a blind EBP, we chose to perform a targeted CT-guided EBP. It involved a CT-guided injection of 10 mL of autologous blood with 2.5 mL of iodinated contrast targeted at the D2-D3 epidural space, with a satisfactory blood spread into the epidural space between C1 and D6 (Figure 3).

On the day after the targeted EBP was carried out, there was a pattern change in the headache, with a worsening in the supine position, which was suggestive of intracranial hypertension. An urgent CT-scan did not show any new findings, indomethacin was prescribed, and the patient was asymptomatic after three days of treatment. Brain MRI performed one month later revealed a complete resolution of the imagiological features and the patient remained without headache recurrence after 30 months.

## 3. Discussion

SIH is increasingly being recognized as a cause of new daily persistent headache with an estimated annual incidence of 5/100,000 and an incidence peak of around 40 years of age [1, 2].

It is due to spontaneous spinal CSF leaks from a tear in the dural membrane, which occurs most often at the reflection of the pia mater to dura mater at the root exit zones, mostly in the cervical region or cervicothoracic junction, although other possible sites are the thoracic, lumbar, and even sacral regions and occasionally the vestibular system, the cribriform plate, or the pituitary fossa [3, 4]. However, a definite location of the leak is not precisely established in a significant number of cases [4]. In a case series of 56 patients with SIH the locations of CSF leakage were cervical in 6 patients, cervicothoracic in 15, mid-lower thoracic in 8, lumbar in 10, and unknown in 17 [5].

Spontaneous spinal CSF leaks probably have a multifactorial etiology, with patients with underlying disorders of the connective tissue having an increased risk of SIH and some cases being preceded by minor trauma, such as coughing, falling, or sports activities [1]. A wide variety of dural defects identified during surgery have been described, such as simple dural holes, meningeal diverticula, and absence of the dura normally covering the spinal nerve root [6].

The classic manifestation of SIH is an orthostatic headache that usually starts within seconds to minutes after assuming an upright position and is relieved following recumbency. A wide range of other neurological symptoms have been described in SIH. More than half of the patients present with posterior neck pain or stiffness, nausea, or vomiting. Between 10% and 50% of patients complain of changes in hearing and a disturbed sense of balance. Less than 10% have visual blurring, visual field defects, diplopia, facial numbness, facial pain, facial weakness, facial spasm, dysgeusia, parkinsonism, or ataxia. Cerebellar haemorrhage and posterior fossa subdural hygroma, dementia, interscapular pain, quadriplegia, upper extremity radicular symptoms, hyperprolactinemia, and galactorrhea have also been described [1, 2, 6–8].

The clinical features of our patient, orthostatic headache accompanied by nausea, vomiting, photophobia, binocular diplopia, and neck stiffness, were very suggestive of this condition.

Brain MRI is important in the evaluation of patients when SIH is suspected. Diffuse pachymeningeal enhancement, a decrease in ventricles size, cerebellar tonsil descent, and subdural fluid collections are findings consistent with SIH, as described in our patient. Spinal MRI may show extra-arachnoid CSF collection, meningeal diverticula, pachymeningeal enhancement, epidural venous plexus engorgement, and bone abnormalities, which may have contributed to the dural tear. Imagiological techniques such as CT myelography or MRI myelography may be used to confirm the diagnosis and to document the site of the leak. Nuclear cisternography and myelography have also been used in the past to confirm the diagnosis [1, 2, 7].

The initial recommended approach includes bed rest, analgesia, caffeine administration, and hydration, although there is no class A or B evidence for this approach [1, 2]. When this conservative treatment fails, an EBP is considered the mainstay of treatment for SIH [1, 4, 7]. The proposed mechanisms of action for an EBP include the formation of a dural tamponade sealing the leak, the restriction of the CSF flow interfering with CSF absorption, and the change of dural resistance and stiffness [6].

The blind lumbar EBP procedure consists of the injection of 10 to 20 cm^3^ of autologous blood, [2] followed by putting the patient in the Trendelenburg position for 30 to 60 minutes, either supine, prone, or lateral, according to the location of the CSF leak. This will allow blood to travel over many spinal segments toward the site of the leak and will maximize the efficacy of this procedure [6]. The reported success rate is 36.8 to 89.5% after the first attempt [5, 9–11]. However, if necessary, a blind lumbar EBP can be repeated, with injection of 20 to 40 cm^3^ of blood, which may be beneficial in an additional 20 to 33% of patients [2]. Some authors recommend a minimum of 5 days between blood patches because local pain or radiculopathy may occur due to the high volume of blood injected [6].

When a repeated lumbar EBP is ineffective, a targeted EBP may be tried. It requires locating the spinal fluid leak and subsequent epidural injection of autologous blood at that level [4, 6, 7, 9]. Although only few reports of targeted CT-guided EBP are described in the literature, they appear to be more efficacious than blind lumbar patches [4], with relief of the symptoms after the first attempt in 71 to 87% of the cases reported [5, 9, 12].

In most reported studies, a blind lumbar EBP was used in the treatment of SIH with thoracic or cervical leak sites because it is easier, and a targeted CT-guided EBP at thoracic or cervical levels was considered to be associated with a higher risk of procedural complications [4, 9, 13]. However, increasing reports show that the blind lumbar EBP is frequently unsuccessful in the treatment of cervical leaks, since an adequate volume of blood for patching the cervical leak is difficult to achieve with a lumbar injection. In some of these cases, a targeted EBP was required [13–23]. A nonrandomized retrospective series evaluated the efficacy of a blind and targeted EBP after a single procedure in a population of 56 SIH patients, and there was a significant difference in the results between the two methods. While the blind blood patch was successful in only 52% of the patients, the targeted EBP achieved a success rate of 87.1% [5].

The classical EBP is considered to be a safe procedure, and complications are usually mild and transient, such as blood leaks to the subcutaneous tissues, cauda equina syndrome, subdural hematoma, back and radicular pain, and epidural infection [24–26].

With current advances in neuroradiology, imaging-guided procedures in the spine have been used more frequently because they appear to be more effective, safer, and more comfortable for the patient, especially when considering cervical leaks [5, 16]. In the case of our patient, as the CSF leak was located at a cervical level, it was assumed that a blind lumbar EBP was less likely to be successful and a targeted CT-guided EBP was attempted with an excellent outcome and without complications.

Patients who fail to improve after conservative treatment and blood patching should be considered for a percutaneous fibrin glue injection or surgical intervention [1, 2, 7].

Published data regarding the outcomes of patients with SIH is limited, but it is thought that most patients have good outcomes after combining conservative management and an EBP [1, 2, 7, 9].

In conclusion, SIH must be considered in the differential diagnosis of patients presenting with new daily persistent and orthostatic headache. In this report we present the targeted CT-guided EBP as an alternative in patients who are refractory to conservative treatment and the blind EBP. It seems to be a safe, minimally invasive, and highly effective treatment, which may avoid the need for a more aggressive approach, such as surgical intervention, in refractory patients.
